# Vimentin inhibits α-tubulin acetylation via enhancing α-TAT1 degradation to suppress the replication of human parainfluenza virus type 3

**DOI:** 10.1371/journal.ppat.1010856

**Published:** 2022-09-15

**Authors:** Pengfei Liu, Shengwei Zhang, Jingyi Ma, Dongning Jin, Yali Qin, Mingzhou Chen

**Affiliations:** 1 State Key Laboratory of Virology and Modern Virology Research Center, College of Life Sciences, Wuhan University, Luo Jia Hill, Wuhan, China; 2 School of Laboratory Medicine, Hubei University of Chinese Medicine, Wuhan, China; 3 Taikang Center for Life and Medical Sciences, Wuhan University, Wuhan, China; University of Southern California, UNITED STATES

## Abstract

We previously found that, among human parainfluenza virus type 3 (HPIV3) proteins, the interaction of nucleoprotein (N) and phosphoprotein (P) provides the minimal requirement for the formation of cytoplasmic inclusion bodies (IBs), which are sites of RNA synthesis, and that acetylated α-tubulin enhances IB fusion and viral replication. In this study, using immunoprecipitation and mass spectrometry assays, we determined that vimentin (VIM) specifically interacted with the N–P complex of HPIV3, and that the head domain of VIM was responsible for this interaction, contributing to the inhibition of IB fusion and viral replication. Furthermore, we found that VIM promoted the degradation of α-tubulin acetyltransferase 1 (α-TAT1), through its head region, thereby inhibiting the acetylation of α-tubulin, IB fusion, and viral replication. In addition, we identified a 20-amino-acid peptide derived from the head region of VIM that participated in the interaction with the N–P complex and inhibited viral replication. Our findings suggest that VIM inhibits the formation of HPIV3 IBs by downregulating α-tubulin acetylation via enhancing the degradation of α-TAT1. Our work sheds light on a new mechanism by which VIM suppresses HPIV3 replication.

## Author summary

Human parainfluenza virus type 3 (HPIV3) is one of the most common pathogenic respiratory RNA viruses, and its replication sites, known as inclusion bodies (IBs), which are formed by nucleoprotein (N) and phosphoprotein (P), are critical in viral RNA synthesis. Exploration of the mechanisms by which viral inclusion bodies are formed in cells may help to identify new antiviral approaches. Our aim was to identify cellular proteins with regulatory effects on the formation of viral IBs. The formation of HPIV3 IBs relies on acetylated α-tubulin, and we found that VIM negatively regulated α-tubulin acetylation by enhancing the degradation of α-TAT1, thereby suppressing the fusion and maturation of viral IBs. Based on the interaction between IBs and VIM, the peptide derived from amino-acid residues 61 to 80 in the VIM head region exerted antiviral effects, providing a new direction for our antiviral study.

## Introduction

Infectious respiratory viral diseases pose a great threat to human health globally. The human parainfluenza virus type 3 (HPIV3) is the most pathogenic HPIV, causing lower respiratory tract infections in children, infants, and people with weakened immunity [1,2]. HPIV3 belongs to the *Paramyxovirus* family, and its viral particles have a negative-strand RNA genome and six structural proteins, including the nucleocapsid protein (N), the phosphoprotein (P), the matrix protein (M), the fusion protein (F), the hemagglutinin and neuraminidase (HN), and the RNA-dependent RNA polymerase (L) [3]. The HN and F proteins contribute to the binding and fusion of HPIV3 with the cell membrane [4]. The N protein, L protein, P protein, and the negative RNA genome form viral replication and transcription complexes [3,5]. We have shown that the co-expression of N and P proteins in cells can lead to the formation of viral factories called inclusion bodies (IBs), in which viral RNA is synthesized [6]. Many studies have reported on viral IBs or viral factories (also referred to as viral condensation and viral replication compartments). Although the names are different, these are all descriptions of viral replication sites [7]. Knowing which cellular proteins can regulate the function of viral inclusion bodies is of great significance for the discovery of antiviral mechanisms. Therefore, our aim was to study the way in which host cells control the replication of HPIV3 and how key cellular proteins can suppress or facilitate viral proliferation.

In previous studies, we showed that HPIV3 IBs relied on the acetylation of cellular α-tubulin for fusion and maturation [8]. We also found that α-tubulin acetylation transferase 1 (α-TAT1) facilitated the replication of HPIV3. However, we wanted to determine whether there was a factor that could suppress the replication of HPIV3. We used immunoprecipitation and mass spectrometry to search for proteins existing in the HPV3 N–P complex, and identified many proteins, including VIM.

The cytoskeleton primarily includes microtubules (MTs), intermediate filaments (IFs), and microfilaments (MFs), which have been studied as common cellular factors in the regulation of the viral life cycle [9–12]. They play important roles in viral adsorption, viral entry, and viral factory formation, assembly and budding, etc. [13–16]. Being among the most abundant protein components in cells, cytoskeletal proteins are responsible for many basic cellular functions, and the mechanisms that cytoskeletal proteins use to regulate viral infection merit further study. Investigating how cytoskeletal proteins participate in the life cycles of viruses could facilitate our understanding of viral infection and assist in the identification of new antiviral therapies.

VIM is the one of the most abundant intermediate filament (IF) proteins, belonging to the type 3 IF cytoskeleton. VIM proteins are involved in the attachment, entry, replication, etc., of viruses [15]. SARS-CoV-2 and Enterovirus A71 both use membrane-located VIM as a binding receptor for entry to cells [17,18]. IAV releases genomes from acidized endosomes using VIM [19,20]. The nonstructural protein 3A of the foot-and-mouth disease virus (FMDV) can directly interact with VIM and maintains the viral replication, but the overexpression of VIM suppressed the viral replication, whereas VIM knockout promoted virus infection [21]. Dengue virus infections result in VIM network rearrangement, which promotes viral replication [22]. These results suggest that VIM plays multiple roles in viral infection. Interestingly, VIM is transported via the microtubule system and interacts with the microtubules, adjusting their polarity and stability [23]. Because the role of VIM in HPIV3 infection is unknown, the interplay between the microtubule system and VIM led us to further investigate whether VIM played a specific role in the HPIV3 replication process.

In this study, we report that the mechanism by which VIM inhibits the replication of HPIV3. Firstly, we found that VIM was a component of the N–P complex and interacted with it through its head domain. A VIM mutant without the head domain was unable to suppress the replication of HPIV3. Secondly, we found that VIM also interacted with α-TAT1, which enhanced its degradation and downregulated the acetylation of α-tubulin, thus suppressing the fusion and maturation of HPIV3 IBs. Thirdly, the amino-acid residues 61–80 in the VIM head domain played a key role in the interaction between N–P and VIM, and a peptide derived from these amino acid residues could inhibit the replication of HPIV3. In this study, we found that VIM negatively regulated the acetylation of α-tubulin, inhibiting the fusion and maturation of HPIV3 IBs, revealing a new antiviral mechanism for VIM, and we identified a peptide with antiviral activity from the interaction site of the VIM and N–P complex.

## Results

### VIM interacts with HPIV3 N–P complexes and suppresses viral infection

**Fig 1:**
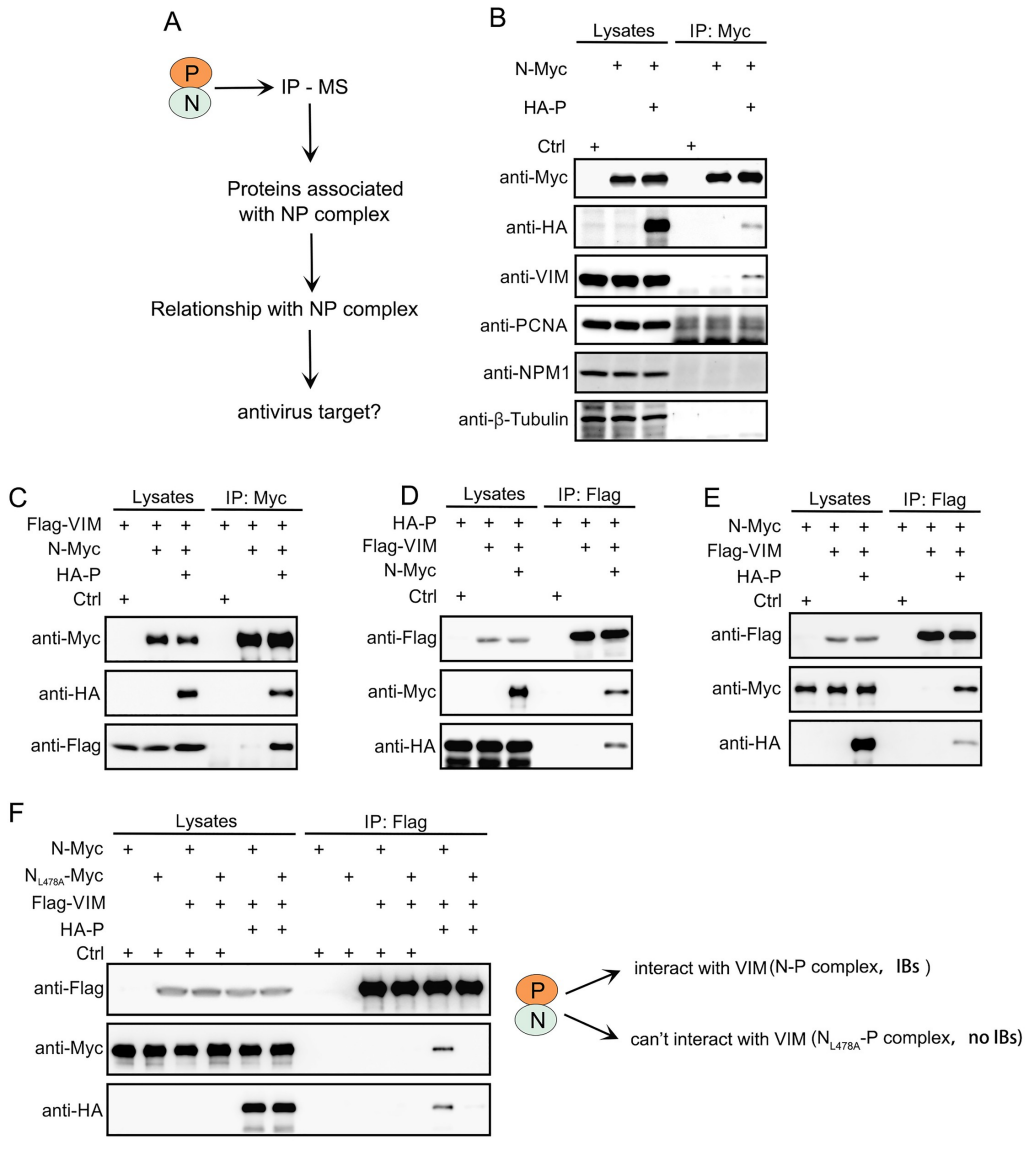
VIM specifically interacts with HPIV3 N–P complex. (A) Experimental design flow chart. HPIV3 N–P complex expressed in HEK293T cells was immunoprecipitated (IP) using anti-c-Myc tag affinity gel. The bands of interest in silver staining were isolated and analyzed using mass spectrometry (MS). (B) Verification of protein interaction in IP/MS results. HEK293T cells were transfected with plasmids encoding P and N alone or in combination, and 48 hours later, cells were harvested and the lysates were subjected to IP for the detection of cellular VIM, PCNA, NPM1, and β-tubulin using specific antibodies. The N–P complex was detected by the expression of N and P proteins. (C, D, E) Confirmation of the interaction between VIM and the N–P complex using IP. HEK293T cells were used to express these proteins. (C) The N–P complex specifically interacted with Flag-VIM. Flag–VIM was transfected with an N-Myc plasmid, or with N-Myc and HA-P plasmids. The individual transfection of Flag–VIM was used as a control. Anti-c-Myc affinity gel was used for immunoprecipitation. (D) Flag–VIM specifically interacted with the N–P complex. HA–P plasmid was transfected with Flag–VIM plasmid, or with Flag–VIM and N-Myc plasmids. The individual transfection of HA–P plasmid was used as a control. The anti-FLAG tag affinity gel was used for immunoprecipitation. (E) Flag–VIM specifically interacted with the N–P complex, but not with the N protein alone. N-Myc plasmid was transfected with Flag–VIM plasmid or Flag–VIM and HA-P plasmids. The individual transfection of N-Myc plasmid was used as a control. Anti-FLAG tag affinity gel was used for immunoprecipitation. (F) The N_L478A_-P complex could not interact with VIM. The HEK293T cells were transfected with N-Myc (or N_L478A_) and Flag–VIM plasmids or with Flag–VIM and HA-P plasmids. Individually transfected N-Myc (or N_L478A_) was used as a control.

To investigate N–P-complex-associated cellular proteins and their functions in the viral lifecycle, we enriched the N–P complexes and then identified interacting proteins and bound cellular proteins using immunoprecipitation (IP) and mass spectrometry, respectively (Fig 1A). The more abundant proteins were considered valuable interactors. Subsequently, we validated the interaction of cellular vimentin (VIM), NPM1, PCNA, and β-tubulin with the N–P complexes using IP. We found that VIM specifically interacted with the N–P complex (Fig 1B). Then, we expressed FLAG-tagged VIM with N, P, and N–P, respectively, and found that only the N–P complex interacted with VIM. Neither N nor P alone could do this (Fig 1C, 1D and 1E), which may be explained by our previous finding that the N–P complex could form IBs, which are the sites of RNA synthesis for HPIV3 in cells; N_L478A_, a mutant of N, could interact with P but could not form IBs with P [6]. To explore whether the interaction between the N–P complex and VIM was due to the formation of IBs, we also examined the interaction between the N_L478A_-P complex and VIM, and found that the N_L478A_-P complex failed to interact with VIM (Fig 1F), which suggested that IBs formed by the N–P complex are critical for the interaction with VIM. In order to confirm the above results, we expressed the N–P complex and VIM protein separately, and then mixed the lysates of the expressed N–P complex and VIM in vitro and performed IP analysis. We could no longer detect the interaction between the N–P complex and VIM protein in the mixture in vitro (S1 Fig), which further suggested that the IBs formed by the N–P complex indeed contributed to the interaction with VIM.

**Fig 2:**
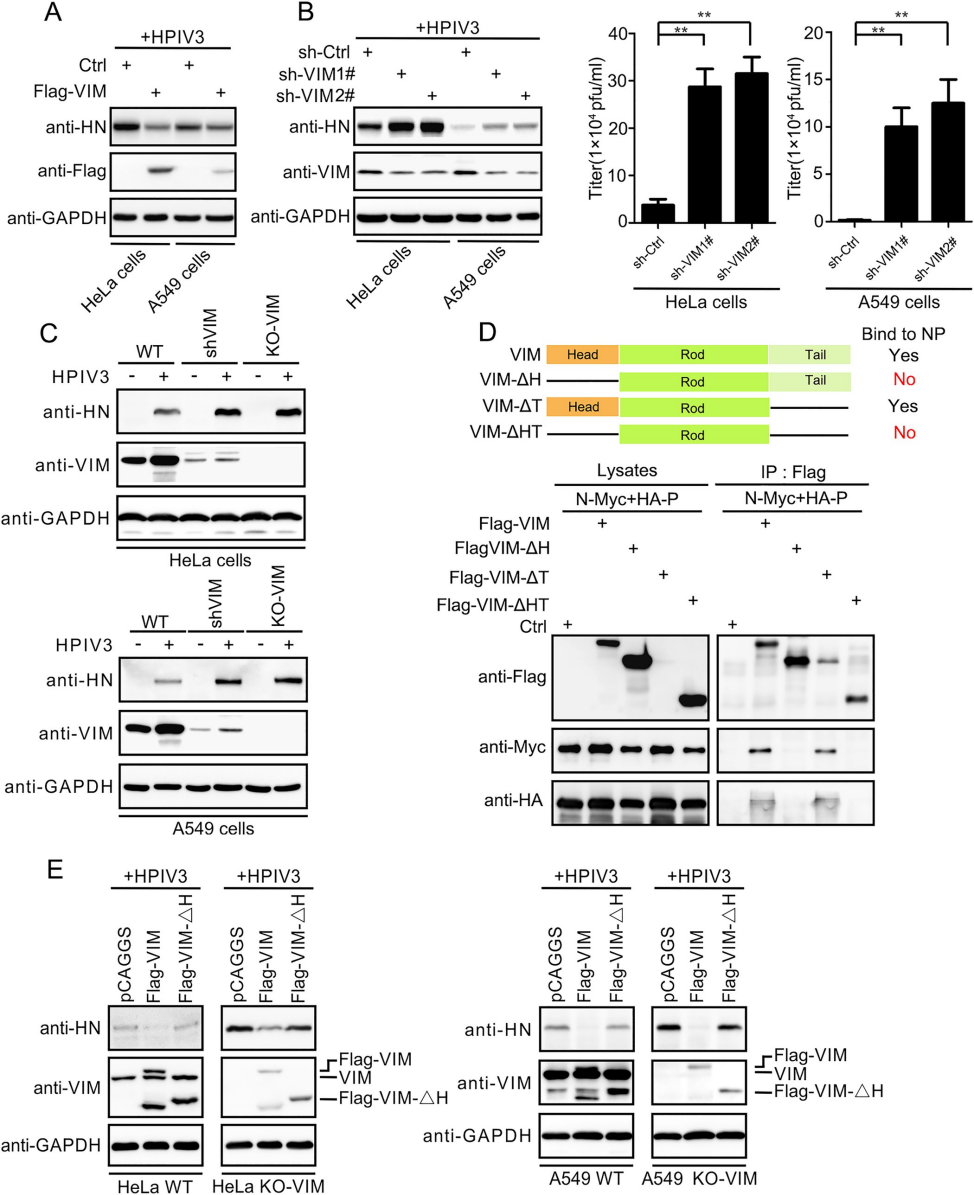
VIM suppresses HPIV3 via its head domain, which is critical for interaction with the N–P complex. (A) Overexpression of VIM suppressed HPIV3 infection. Flag–VIM plasmid was transfected into HeLa and A549 cells prior to HPIV3 infection (MOI = 0.1). HPIV3 HN and GAPDH proteins were detected using Western blotting. (B) VIM-KD promoted HPIV3 infection. VIM was knocked down using sh-VIM1# and sh-VIM2# in HeLa and A549 cells prior to HPIV3 infection (MOI = 0.1). The HPIV3 HN protein was detected. Virus titers were measured using a plaque assay. (C) KO of VIM promoted HPIV3 infection. Wild-type (WT), stable VIM-KD (sh-VIM1#), and stable VIM-KO cells were used to detect HPIV3 infection levels. HPIV3 HN protein was detected. (D) Schematic diagram of wild-type VIM and VIM mutants (VIMΔH, VIMΔT, and VIMΔHT). VIMΔH and VIMΔHT could not interact with the N–P complex. Flag–VIM and VIM mutant plasmids were transfected into HEK293T cells with N and P plasmids. Cell lysates were immunoprecipitated using anti-FLAG tag affinity gel. (E) VIMΔH expression could not suppress HPIV3 replication in VIM-KO and WT cells. VIM and VIMΔH were overexpressed in WT/VIM-KO cells, followed by HPIV3 infection (MOI = 0.1). The HPIV3 HN protein was detected. Values are means ± SDs from three experiments. Student’s t test: * p value<0.05, ** p value<0.01, *** p value<0.001, **** p value<0.0001, and ns = not significant.

Because IBs are the replication sites of HPIV3 and VIM interacts with IBs, we thought that VIM might be involved in the replication of HPIV3. Therefore, we overexpressed VIM in HeLa and A549 cells, which inhibited the replication of HPIV3 (Fig 2A). Then, we knocked down the expression of VIM and found that viral HN protein and the titer of the virus increased significantly (Fig 2B). To confirm that VIM inhibited viral replication, we used CRISPR-Cas9 to knockout VIM and constructed HeLa and A549 cell lines with VIM stably knocked out (S2 Fig). This significantly enhanced the viral replication, similar with the effect obtained from the knockdown of VIM (Fig 2C).

To map the region of VIM interacting with the N–P complex, we constructed three deletion mutants of VIM—VIM deleted head domain (VIMΔH), VIM deleted tail domain (VIMΔT), and VIM deleted head and tail domain (VIMΔHT)—and then examined the interactions of the three mutants with the N–P complex. We found that the deletion of the head domain abrogated the interaction of VIM with the N–P complex (Fig 2D), indicating that the head domain mediated the interaction with the N–P complex.

Because the head region of VIM contributed to the interaction between VIM and the N–P complex, we wanted to determine whether the head region of VIM was also involved in the inhibition of the viral replication. Thus, we overexpressed VIM and VIMΔH in wild-type and VIM-knockout cells, respectively; the expression of VIM inhibited the replication, but VIMΔH did not (Fig 2E), suggesting that VIM suppressed the replication of HPIV3 via its head domain.

### VIM suppresses the replication of HPIV3 by blocking the fusion of IBs

**Fig 3:**
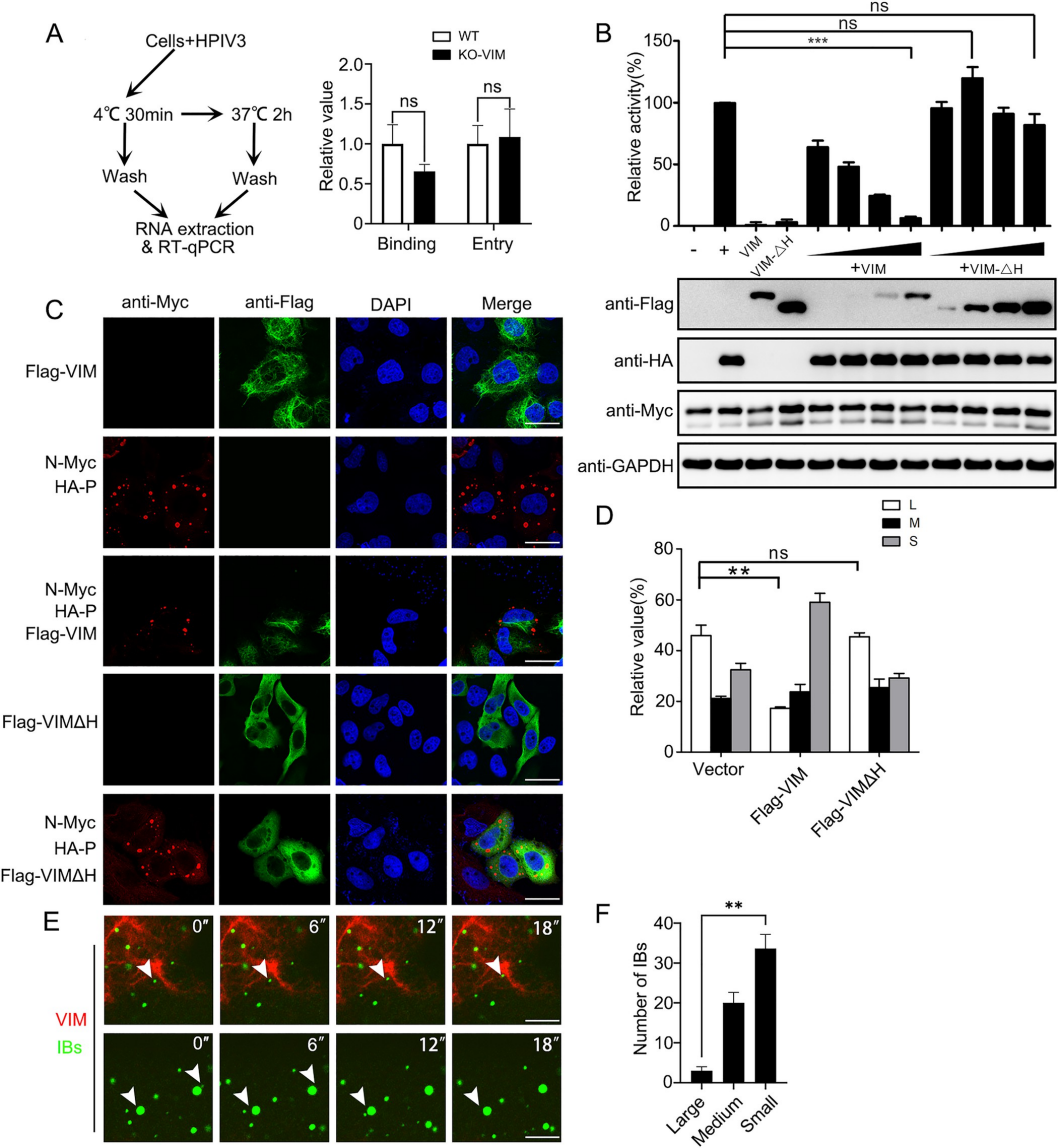
VIM suppresses HPIV3 replication and inhibits formation of HPIV3 IBs. (A) VIM did not impair the binding and entry of HPIV3. Schematic diagram of HPIV3 binding and entry assay in WT/VIM-KO A549 cells. Results for HPIV3 RNA levels were obtained using RT-qPCR, and the ratios of the relative changes normalized to the control group are shown. Three technical replicates were carried out in each analysis. At least three replicate experiments were performed. (B) VIM suppressed HPIV3 replication. An HPIV3 minigenome (MG) activity assay showed that VIM suppressed HPIV3 MG activity in a dose-dependent manner, but VIMΔH did not. HeLa cells infected with VTF7-3 were transfected with plasmids encoding N, P, L, and viral MG. “-” means that only P plasmid was not transfected; “+” means that N, P, L, and MG plasmids were all transfected. VIM and VIMΔH were both transfected in the “-” and “+” groups, to exclude their regulation of HPIV3 MG activity. Both protein expression and HPIV3 MG relative activity were detected. The activity of the “+” group was shown as 100% activity. (C) VIM suppressed the formation of HPIV3, but VIMΔH did not. Flag–VIM and Flag–VIMΔH were transfected alone or co-transfected with N-Myc and HA–P complex in HeLa cells, and an immunofluorescence assay was conducted. N-Myc was considered a component of IBs and indicated the location and sizes of IBs. Mouse anti-c-Myc antibody and rabbit anti-FLAG antibody were used. Scale bar: 20 μm. (D) The IB sizes were divided into large, medium, and small, and statistical analysis was carried out. IBs in three virus-infected fields were analyzed. Values are means ± SDs. (E) Live cell imaging showed that VIM suppressed the fusion of HPIV3 viral IBs. HeLa-GFP–P cell lines were infected with HPIV3, and mCherry–VIM was transiently expressed. GFP–P-labeled IBs adjacent to VIM are shown in the upper row, and IBs in non-VIM regions are shown in the lower row. The white arrows indicate IBs adjacent to VIM in the upper row and the IB fusion process in the lower row. Scale bar: 5 μm. (F) The IB sizes were divided into large, medium, and small, and statistical analysis was carried out. IBs adjacent to VIM in three areas were counted as shown in the S1 Movie. Values are means ± SDs. For panels A, D and F, Student’s t test was used with * p value<0.05, ** p value<0.01, *** p value<0.001, **** p value<0.0001, and ns = not significant.

It has been reported that VIM is involved in the life cycle of the virus, including by binding to viral surface proteins, facilitating the viral entry, or by interacting with viral structural/nonstructural proteins, regulating the viral replication [24]. To clarify how VIM inhibited the viral replication, we first compared the virus binding and entry abilities in wild-type and knockout VIM cells, and the results showed that the knockout of VIM did not affect the viral binding and entry (Fig 3A). Because the mini-genome replicon of HPIV3 can specifically detect the synthesis of viral RNA [6], we used the HPIV3 mini-genome replicon (MG) to examine whether VIM regulated viral RNA synthesis. HPIV3 N protein, P protein and L protein were expressed with a luciferase fused with viral replication regulation sequences (HPIV3 mini-genome, MG) and the luciferase activity was measured as also a control without P protein, and their ratio was used to quantify viral replication activity (MG activity). The results showed that VIM significantly inhibited the expression of the reporter gene of the mini-genome replicon, but VIMΔH did not (Fig 3B), suggesting that VIM inhibited the viral replication and that the inhibition was mediated through the head domain of VIM. Next, we wanted to discover how VIM inhibited viral RNA synthesis, having previously shown that the fusion of IBs was required for viral RNA synthesis [25]. Therefore, we examined the effect of VIM expression on the morphology of IBs and found that there were significantly fewer large IBs in VIM-expressing cells than in VIMΔH-expressing cells (Fig 3C and 3D), indicating that VIM expression was detrimental to the formation of HPIV3 IBs. To confirm that VIM inhibited IB formation, we used a live cell-imaging system to observe the process of VIM regulating IB fusion under HPIV3 infection. This included the use of HPIV3-infected HeLa cells stably expressing GFP–P to observe the IB fusion process [26], and the expression of mCherry–VIM to observe the effect of VIM on IB fusion. The results showed that the IBs adjacent to VIM were small and few fusions occurred during the observation period, whereas the IBs farther away from VIM were large and the fusion of small IBs to large IBs could be observed (Fig 3E, 3F and S1 Movie).

### VIM blocks the fusion of IBs by inhibiting α-tubulin acetylation

**Fig 4:**
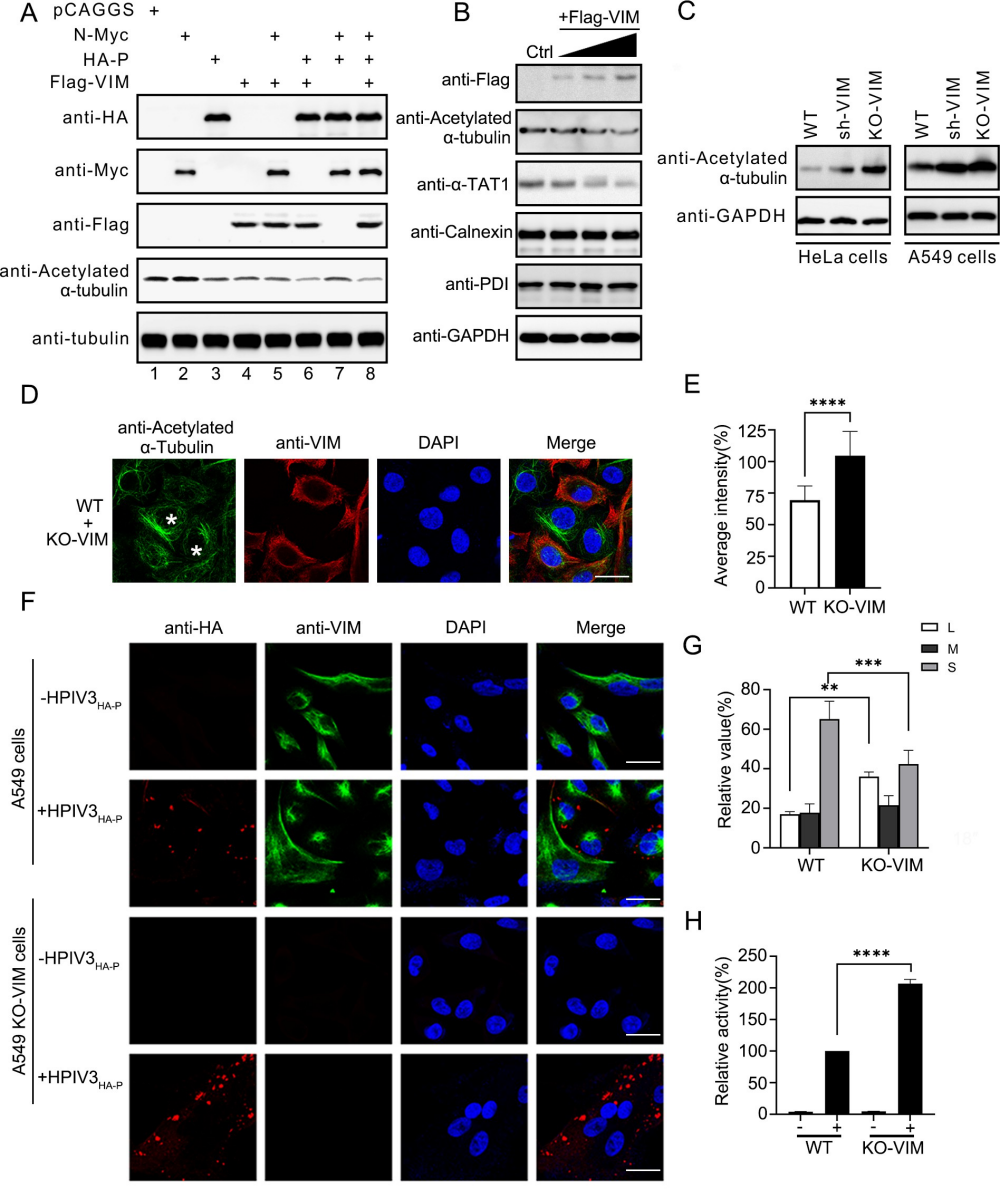
VIM negatively regulates the acetylation of α-tubulin, inhibiting the formation of HPIV3 IBs. (A) VIM downregulated α-tubulin acetylation. N-Myc, HA–P, and Flag–VIM plasmids were transfected alone or together, and the acetylation of α-tubulin was detected as well as the overexpressed proteins. (B) VIM downregulated the α-TAT1 protein level and the acetylation of α-tubulin. The expression of VIM protein in HeLa cells was gradually increased. The protein level of α-TAT1 and acetylation of α-tubulin were detected. Two endoplasmic reticulum proteins, calnexin and PDI, were also detected. (C) Acetylation level of α-tubulin in KD and VIM-KO cells. Acetylation of α-tubulin in WT/VIM-KD/VIM-KO cells (HeLa/A549) was detected. (D) Acetylated α-tubulin morphology in WT and VIM-KO cells. WT A549 and KO-VIM A549 cells were mixed (1:1), and the acetylated α-tubulin was detected using specific antibodies in the IF assay. Scale bar: 20 μm. (E) Fluorescence intensity of acetylated α-tubulin in (D) was analyzed using ImageJ, and the results are shown in the histogram with difference analysis. Ten visible fields of cells were observed and analyzed. (F) HPIV3 viral IBs fused to increase in size in the VIM-KO A549 cells, HPIV3_HA-P_-infected A549 cells, and A549 VIM-KO cells. HPIV3 viral IBs were identified with HA-tagged P protein using the IF assay. Cellular VIM was also observed. Rabbit anti-VIM antibody and mouse anti-HA antibody were used. Scale bar: 20 μm. (G) Inclusion body size statistical chart. HPIV3 IBs were divided into large, medium, and small; the numbers were counted; and statistical analysis carried out. IBs in three virus-infected fields were analyzed. (H) HPIV3 MG activity in VIM WT/KO A549 cells. HPIV3 MG activity assay was applied as previously described, and the relative change is shown. The activity of the “+” group in WT cells was shown as 100% activity. Values are means ± SDs from three experiments. For panels E, G, and H, Student’s t test was used and * p value<0.05, ** p value<0.01, *** p value<0.001, **** p value<0.0001, and ns = not significant.

Because previous studies showed that the acetylation of α-tubulin enhanced the fusion of HPIV3 IBs, and that VIM bound to tubulin and affected its stability [8,27,28], we wanted to discover whether VIM blocked IB fusion by inhibiting α-tubulin acetylation. N, P and VIM were separately or collectively transfected into HeLa cells. α-tubulin acetylation was downregulated by VIM overexpression (Fig 4A, lane 4), and N–P and VIM co-transfection did not restore the α-tubulin acetylation level (Fig 4A, lane 8). The overexpression of the VIM protein decreased the acetylation of the microtubules and the protein level of the α-tubulin acetyltransferase α-TAT1 (Fig 4B). The calnexin, PDI, and GAPDH protein levels were not affected. The α-tubulin acetylation level in the stable-knockdown and knockout VIM cell lines were also upregulated, and the morphology of the acetylated α-tubulin was more marked compared with that in the wild type cells (Fig 4C, 4D and 4E). Then, HPIV3_HA-P_ was used to infect WT/VIM-KO A549 cells and visualize viral IBs by highlighting the location of HA-P [26]. Viral IBs tended to be larger (Fig 4F and 4G), and the HPIV3 MG activity significantly increased in the VIM-KO cells (Fig 4H). In summary, VIM negatively regulated α-tubulin acetylation, inhibiting the formation of viral IBs.

The acetylation of α-tubulin was highly modified by α-TAT1 [29–31], and we also noticed that the overexpression of VIM induced a decrease in α-TAT1 protein levels. Next, we wanted to determine whether VIM enhanced α-TAT1 degradation and examine the possible mechanisms.

### VIM interacts with α-TAT1 and degrades α-TAT1, resulting in the suppression of HPIV3 IB fusion

**Fig 5:**
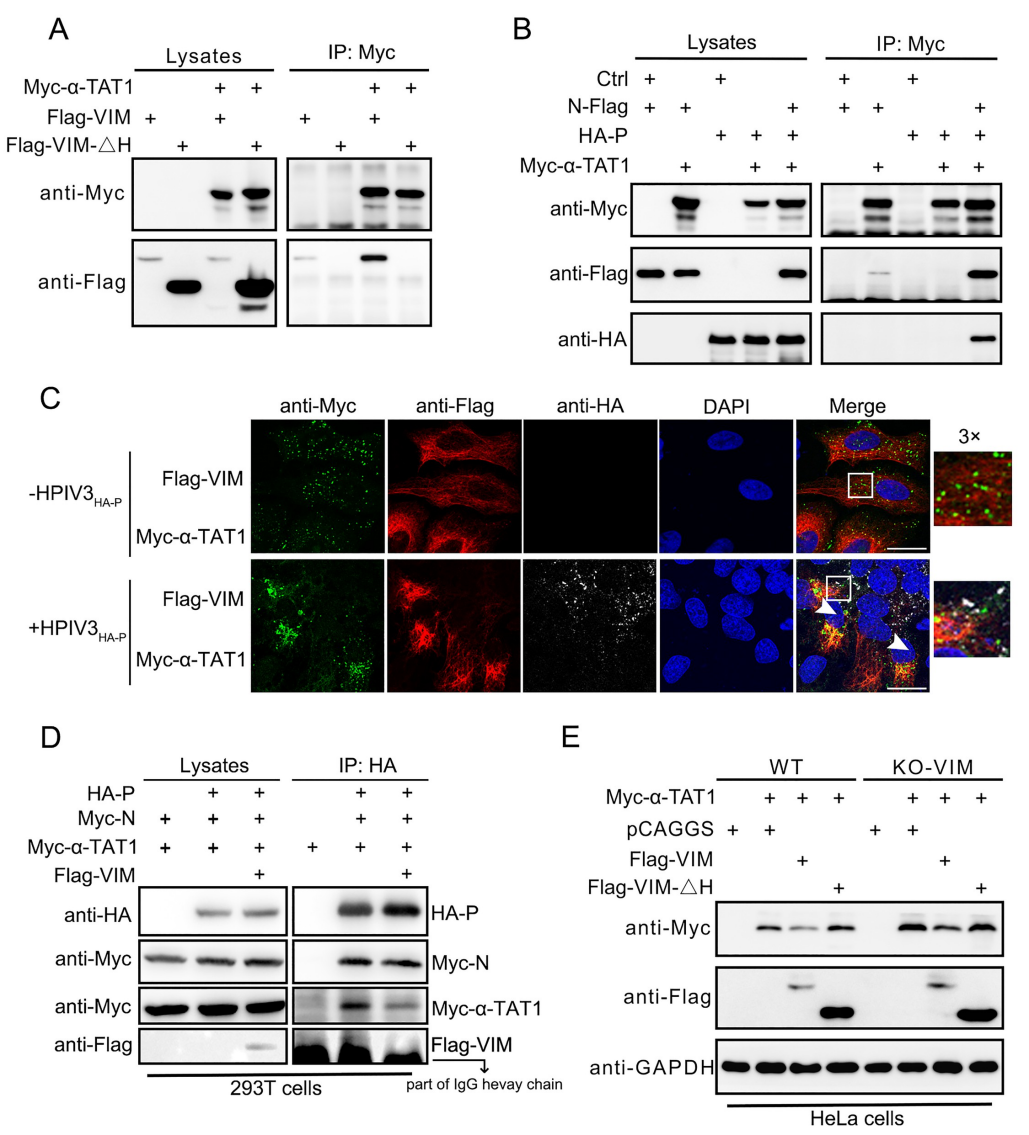
VIM enhances α-TAT1 degradation through its interaction to suppress the formation of HPIV3 IBs. (A) α-TAT1 interacted with VIM but not with VIMΔH. Myc–α-TAT1 plasmid was co-transfected with Flag–VIM plasmid or Flag–VIMΔH plasmid in HEK293T cells. Cell lysates were immunoprecipitated using anti-c-Myc tag affinity gel. (B) α-TAT1 interacted with the N–P complex. Myc-α-TAT1, N-Flag, and HA–P plasmid were transfected in HEK293T cells. As IP controls, Myc–α-TAT1 was co-transfected with N-Flag or HA–P. N-Flag or HA–P were individually transfected in groups. Cell lysates were immunoprecipitated using anti-c-Myc tag affinity gel. (C) Localization of VIM, α-TAT1, and HA–P-labeled viral IBs in HeLa cells. HPIV3_HA-P_ virus was used to show viral IBs. Rat APC-anti-FLAG antibody, mouse anti-c-Myc antibody and rabbit anti-HA antibody were used to detect HA–P. The white arrows show the cells with VIM, IBs, and α-TAT1. Scale bar: 20 μm. (D) HA-P was immunoprecipitated with N-Myc, Myc-α-TAT1 and Flag-VIM expressed in 293T cell lysates. (E) VIM enhanced α-TAT1 degradation, but VIMΔH did not. Flag–VIM or Flag–VIMΔH was transfected with Myc–α-TAT1 in WT/VIM-KO cells, and α-TAT1 protein levels were detected. HeLa and A549 cell lines were used to confirm the degradation.

We transfected α-TAT1 and VIM/VIMΔH, and the results of immunoprecipitation showed that α-TAT1 could interact with VIM but not with VIMΔH (Fig 5A). The N–P complex also interacted with α-TAT1 (Fig 5B). This suggested that both viral IBs and α-TAT1 were regulated by VIM. Using confocal immunofluorescence, we observed that viral IBs forming by HPIV3_HA-P_ located with α-TAT1 and VIM in HeLa cells (Fig 5C). Compared with cells without HPIV3 infection, α-TAT1 co-aggregated to regions where VIM was present. When HPIV3 infected cells, VIM changed location and aggregated (S3A Fig). This phenomenon showed that VIM responded to HPIV3 infection and co-aggregated with α-TAT1. VIM weakened the interaction between N-P complex and α-TAT1(Fig 5D). As VIMΔH did not suppress HPIV3 replication or interact with α-TAT1, we wanted to know whether VIMΔH affected the stability of α-TAT1. In WT and VIM-KO cells, α-TAT1 could be degraded by VIM but not by VIMΔH (Fig 5E). We also used live cell imaging to observe the microtubule, viral IBs, and VIM together, and we found that viral IBs associated with both VIM and the microtubule (S3B and S3C Fig). Using inhibitors targeting autophagy (as well as autophagy activators) and proteasome-related degradation, we initially found that the degradation of α-TAT1 by VIM mainly occurred through the autophagy pathway (S3D Fig). The formation of HPIV3 IBs in α-tubulin acetylation deficiency cells (also KO-VIM) were suppressed (S3E Fig). Together we found that VIM used its head domain to interact with the N–P complex and α-TAT1, downregulating the acetylation level of α-tubulin and inhibiting IB fusion. The interaction between the VIM N–P complex and α-TAT1 is important in the inhibition of HPIV3 replication by VIM.

### A peptide derived from the interaction site of VIM and N–P complex exhibits antiviral ability

**Fig 6:**
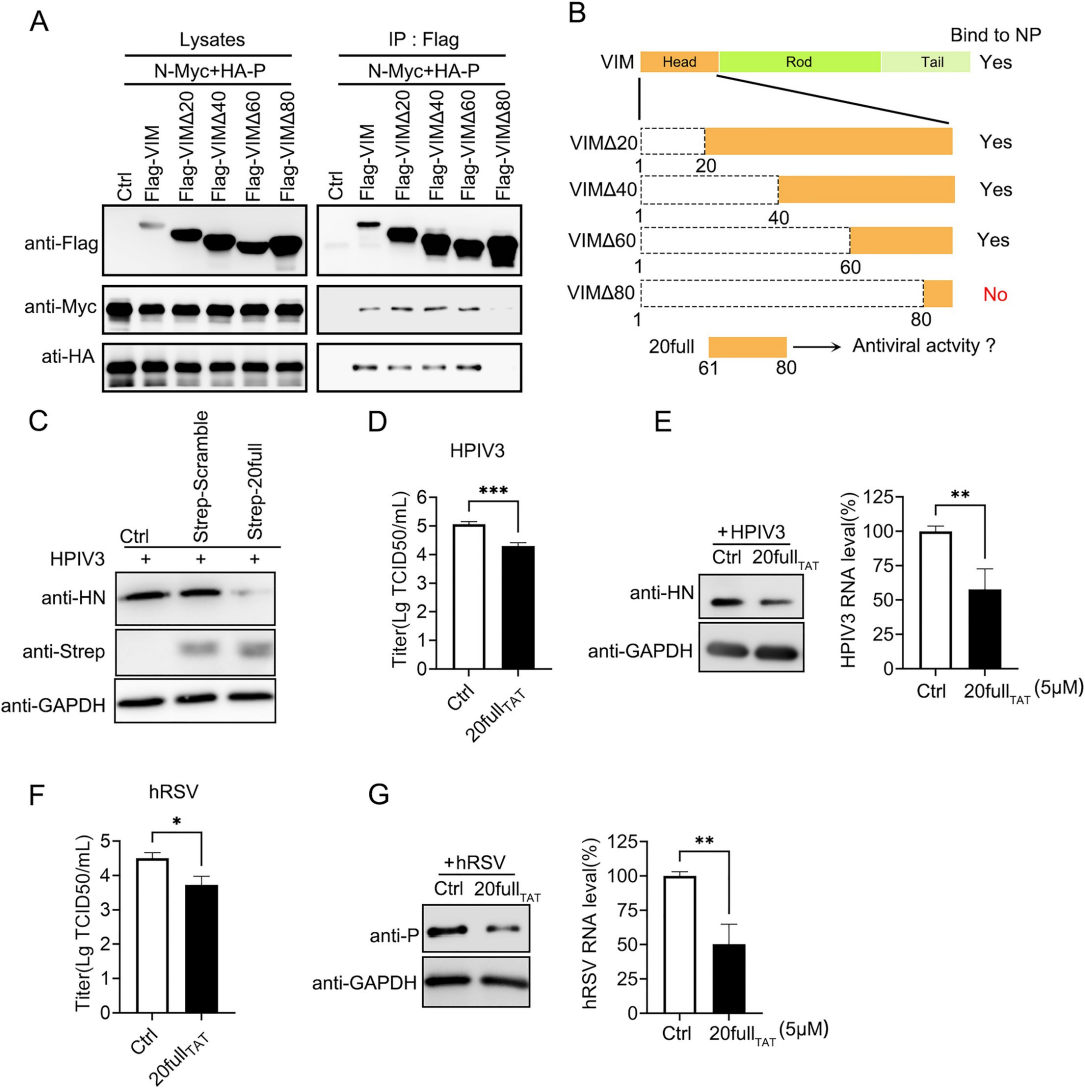
A peptide from the interaction site of VIM and N–P complex exhibits antiviral ability. (A) Interaction between VIM mutants and the N–P complex. VIM head domain-related mutant (VIMΔ20, VIMΔ40, VIM60, and VIMΔ80) plasmids were transfected into HEK293T cells with N-Myc and HA–P plasmids. The cell lysates were subjected to an IP assay using anti-FLAG affinity gel. Lane 12 shows that the interaction between the N–P complex and VIMΔ80 mutant was lost. (B) Graphical representation of the results of (A). (C) Strep-20 full inhibited HPIV3 infection. Strep-20 full plasmid or strep-scramble plasmid was transfected into A549 cells prior to HPIV3 infection (MOI = 0.1). Cells underwent lysis, and HPIV3 HN protein, strep-tagged proteins, and GAPDH were detected. (D) Titer of HPIV3 in supernatants from HPIV3 infected A549 cells which were treated with 20 full_TAT_ peptide, and TAT was used as a control. (E) 20 full_TAT_ peptide inhibited HPIV3 replication. A549 cells were infected with HPIV3 before being treated with 20 full_TAT_ (HPIV3 MOI = 0.1). Both HPIV3 HN protein and viral RNA levels were detected. (F) Titer of hRSV in supernatants from hRSV infected A549 cells which were treated with 20 full_TAT_ peptide, and TAT was used as a control.(G) The 20 full_TAT_ peptide inhibited hRSV replication. A549 cells were infected with hRSV (MOI = 1) before being treated with 20 full_TAT_ peptide and TAT peptide. Viral P protein and RNA levels were detected. Values are means ± SDs from three experiments. For panels D, E, F, and G, Student’s t test was used, and * p value<0.05, ** p value<0.01, *** p value<0.001, **** p value<0.0001, and ns = not significant.

Earlier studies concerning the interacting components of viral proteins showed that polypeptides derived from these interacting proteins exhibited antiviral activity [32–34]. With regard to the important role of the VIM head domain in the inhibition of the replication of HPIV3, we next tried to pinpoint the part of the domain participating in its interaction with the N–P complex. From the first amino acid, every 20 amino acids were deleted, and four mutants of VIM were constructed (Fig 6A and 6B). The VIM mutant with amino acids 1–80 deleted could not interact with the N–P complex, but the mutant with amino acids 1–60 deleted, and others, still showed the interaction (Fig 6A). We refer to the sequence of amino acids 61–80 as 20 full (Fig 6B). To validate the results, a fusion protein including mCherry, amino acid residues 61–80, and a strep tag were constructed into one plasmid in order, along with a scramble control plasmid. These two plasmids were named strep-20 full and strep-scramble. Strep-20 full and strep-scramble were transfected into A549 cells prior to HPIV3 infection, and the viral protein level was only suppressed by strep-20 full (Fig 6C). 20 full peptide with a FAM fluorescent group named as FAM-20 full peptide was used to confirm that the peptide could be loaded into cells with the help of TAT, which is a widely used cell-penetrating peptide (S4A Fig, first row). In the HeLa cells infected with HPIV3_HA-P_, the FAM-20 full peptide was also located with HPIV3 IBs (S4A Fig, third row). Cells that were treated only with the TAT peptide showed no signal in the FAM channel, indicating that the signal in the FAM channel was specifically from the FAM-20 full (S4A Fig, second row). The results also confirmed our discovery that the amino acid residues 61–80 facilitated the interaction between VIM and the N–P complex and maintained its antiviral ability.

To confirm the antiviral activity of these amino acids, amino-acid residues 61–80 were synthesized with TAT as one peptide, which was named 20 full_TAT_, to test their antiviral ability. First, the titers of HPIV3 and hRSV were downregulated significantly by the peptide (Fig 6D and 6F). The effects of the peptides on cell viability were verified, and no significant change was observed for the TAT peptide used as a control (S4B and S4C Fig). Next, A549 cells were infected with HPIV3 and hRSV, and then treated with 20 full_TAT_. Both the viral protein and RNA levels were downregulated by the 20 full_TAT_, and the results indicated that this peptide had antiviral ability (Fig 6E and 6G).

## Discussion

In our previous studies, we found that the HPIV3 N–P complex formed viral IBs, promoting viral replication; that this process depended on α-tubulin acetylation catalyzed by α-TAT1; and that acetylated α-tubulin promoted the fusion and maturation of viral inclusion bodies and HPIV3 replication [8]. However, little was known about the way in which cellular proteins regulated the fusion of viral IBs and whether this was related to the regulation of α-tubulin acetylation.

In this study, we showed that cellular VIM interacted with the HPIV3 N–P complex and clarified its roles in the negative regulation of viral replication. We can draw several conclusions from our findings: (1) VIM specifically binds to the N–P complex through its head domain and suppresses HPIV3 infection, and the ability to form viral IBs is important for the interaction (Figs 1 and 2). (2) VIM blocks the N–P complex’s ability to fuse and form viral IBs, and suppresses HPIV3 replication, but not viral binding and entry (Fig 3). (3) VIM enhances α-TAT1 degradation, downregulating the acetylation of α-tubulin, which results in the suppression of the fusion and maturation of viral IBs (Figs 4 and 5). (4) The amino acids in the VIM head region from 61 to 80 are critical for the interaction between VIM and the N–P complex, and a peptide from this interaction site has antiviral activity (Fig 6). Clarifying the proteins that participate in viral replication is complicated. Although some proteins have been identified as components that interact with viral IBs and regulate viral replication, the critical interaction sites were unclear [35,36].

In our study, we performed immunoprecipitation, which showed that the HPIV3 N–P complex, the minimal component for the formation of IBs, interacted with cellular VIM; we then used mass spectrometry to identify the interacting proteins. Then, we further verified and confirmed the interaction of N–P complexes between endogenous and exogenous VIM. N_L478A_, a mutant interacting with P, could not form viral IBs, and was used to express with P. The N_L478A_–P complex could not interact with VIM (Fig 1B and 1D). The results show that the specific interaction between VIM and the N–P complex depends on the ability of the N–P complex to form viral IBs. In cells, we found that the N–P complex formed IBs adjacent to VIM and the microtubule, and we also found that IBs fused to become bigger and farther away from VIM (Fig 3E). Because VIM was transported by microtubules and organized into networks, and HPIV3 inclusion bodies also interact with the microtubule component α-tubulin [8,27], we think that the N–P complex may interact with VIM in the same way as α-tubulin but that the interaction suppresses the formation of viral IBs. The interaction between VIM and microtubules was identified in vitro, but clear interactions between the components of the microtubules have not been identified. We think that the microtubule may provide mechanical force for the formation of viral IBs.

We focused on the VIM protein and how VIM regulated HPIV3 infection. The overexpressed of VIM inhibited HPIV3 infection in both the HeLa and A549 cell lines. Knocking down VIM in the two cell lines increased the HPIV3 titer by about ten times (Fig 2B), and the level of the HPIV3 protein in VIM-KD and VIM-KO cells was similarly improved (Fig 2C). These results indicated that VIM was a negative regulator of HPIV3 infection. Functional domains in VIM were deleted, and only VIMΔH was unable to interact with the N–P complex (Fig 2D). Then, we expressed VIM and VIMΔH in WT/VIM-KO cell lines. We found that VIM still inhibited HPIV3 replication but VIMΔH did not (Figs 2E and 3B). These results showed that VIM negatively regulated HPIV3 infection via the interaction between the head domain and the N–P complex. It was interesting to note that VIM without the head region, the domain that regulates the vimentin fiber’s assembly [37, 38], could neither interact with the N–P complex nor suppress HPIV3 replication. These results suggest that the different mechanical forces from vimentin and the microtubule may regulate the functions of viral replication sites.

We were interested in exploring the relationship between the formation of IBs and the expression of VIM. For some viruses such as EV71, SARS-CoV, and SARS-CoV-2, VIM located in the cell membrane promotes the binding and entry of viruses [17,18,24,39]. Using RT-qPCR, we found that there was no significant difference between WT and VIM-KO cells in terms of HPIV3’s binding and entry (Fig 3A). Then, using the HPIV3 MG reporter system, we found that the overexpression of VIM inhibited the activity of HPIV3 MG but VIMΔH did not (Fig 3B). We also found that IBs tended to be smaller in VIM-overexpressing cells and larger in VIM-KO cells. The results showed that the fusion and maturation of viral IBs were also inhibited by VIM but not by VIMΔH (Figs 3C and 4F). Using live cell imaging, we observed that small HPIV3 IBs adjacent to VIM took longer to fuse with each other than the IBs adjacent to microtubules in the HeLa cells (S3B and S3C Fig, S1 Movie). In summary, VIM suppressed the replication of HPIV3 through its head domain and specifically suppressed the formation of viral IBs. The suppression depends on the interaction, but there may be other proteins participating in the regulation process, because the viral IBs were not all adjacent to VIM (Fig 3D and S1 Movie).

We explored whether another possible regulatory mechanism existed. VIM is also a microtubule-associated protein and assembles into VIM fibers and networks [23]. VIM also interacts with microtubules to template microtubule networks and enhances cell polarity in the process of directional migration [28]. The two cytoskeletons have a close relationship in cells. Based on our results and previous work [8], we hypothesized that VIM may affect the modification of microtubules to regulate the function of IBs after they have detached from VIM. When VIM was overexpressed, the protein levels of acetylated α-tubulin and α-tubulin acetylation transferase 1 (α-TAT1) were downregulated (Fig 4A and 4B). We demonstrated that HPIV3 IBs remodeled the ER membrane and interacted with PI4KB to promote viral replication [40], but we did not find that VIM could change the levels of calnexin and PDI protein (two ER proteins) (Fig 4B). These results show that VIM affected α-tubulin and exclude other known regulatory modes of HPIV3 IB formation.

The acetylated α-tubulin level in the VIM-KD and KO cells was higher than that in the wild-type cells (Fig 4C and 4D). In the VIM-KO A549 cells, HPIV3_HA-P_ formed larger viral IBs (Fig 4F), which proved to be more mature and had higher viral replication activity [6]. These results indicate that VIM enhanced α-TAT1 degradation and suppressed the fusion of viral IBs, and that the regulatory effect of VIM on α-TAT1 was significant. We also found that α-TAT1 could interact with the VIM N–P complex, and that the interaction between α-TAT1 and VIM relied on the VIM head domain (Fig 5A and 5B). In HPIV3_HA-P_-infected cells, VIM gathered with α-TAT1 and located with viral IBs in the nearby regions (Fig 5C). We thought that VIM could interact with both IBs and α-TAT1 to achieve its antiviral effect. VIMΔH could neither suppress HPIV3 replication nor enhance α-TAT1 degradation (Fig 5D and 5E), showing that VIM could downregulate α-tubulin acetylation by enhancing α-TAT1 degradation to suppress HPIV3 replication. We confirmed these results in both wild-type and VIM-KO cells including HeLa and A549 cells. In summary, we revealed the relationship between α-tubulin acetylation and VIM expression, and proved that the absence of VIM improved the α-tubulin acetylation level and promoted the fusion of HPIV3 IBs, resulting in improved HPIV3 replication.

We mapped the region of the VIM head domain involved in the interaction between VIM and the N–P complex. When VIM lost amino acids 1–80, it could not interact with the N–P complex, but VIM without amino acids 1–60 could still interact (Fig 6A). This suggested that the amino-acid residues 61–80 formed the key area for the interaction between VIM and the N–P complex. The fusion proteins, strep-20 full, expressed in A549 prior to HPIV3 infection, could inhibit the HPIV3 protein level, which suggested that 20 full had an antiviral effect (Fig 6C). Furthermore, we treated cells with 20 full_TAT_ prior to HPIV3 and hRSV infection. The results showed that the levels of viral proteins, titers and RNA were downregulated by the treatment with 20 full_TAT_ (including HPIV and hRSV), which indicated that 20 full_TAT_ had an antiviral effect on the replication of HPIV3 and hRSV (Figs 6D–6G, S4C and S4D). Because both hRSV and HPIV3 could form viral factories, we considered that the peptide might affect the functions of their viral factories. With regard to FAM-20full, this located with the HPIV3 IBs but also partly in the nucleus (S4A Fig). We think this might be related to the infection of HPIV3, because the peptide was not present in the nuclei of cells without viral infection. If we could optimize the peptide to make it present in the cytoplasm only, together with the viral IBs, it might exhibit stronger antiviral activity. On the other hand, because the peptide was derived from cellular vimentin, it might target other cellular proteins with unknown effects, and this should be further studied to gain more specific knowledge about the antiviral activity of the peptide. In summary, we identified a peptide from the VIM/N–P complex interaction site and verified its antiviral effect.

In this study, we have shown that HPIV3 IBs recruited acetylated α-tubulin and PI4KP to promote viral replication [8,40]. Although we did not find HPIV3 IBs directly located with α-TAT1, an interaction between the N–P complex and α-TAT1 did exist (Fig 5B and 5C). This may be because the use of α-TAT1 by IBs does not require colocalization, in contrast to the recruitment of PI4KP by IBs. VIM could interact with α-TAT1 and the N–P complex, and enhanced α-TAT1 degradation, downregulating α-tubulin acetylation. Because we found that VIM, α-TAT1, and IBs localized in adjacent cell areas in HPIV3-infected cells, we concluded that VIM responded to HPIV3 infection and changed its location.

In a study on the HCMV, a DNA virus that replicates in the nucleus, it was demonstrated that α-TAT1 enhanced α-tubulin acetylation, promoting the rotation of the nucleus, and spatially segregated viral DNA from inactive histones and host DNA in the nucleus, thus maximizing the replicative ability of the virus [41]. MPyV, a mouse DNA virus, uses its capsid VP1 protein to stimulate α-TAT1 activity, leading to microtubule stabilization and promoting viral replication [42]. Because RNA viruses such as HPIV3 mainly replicate in cytoplasm (although viruses such as IAV replicate in the nucleus), it is possible that the intercellular cytoskeletal crosstalk also affects viral replication, but whether the mechanism of these viruses in maximizing their replication is related to α-TAT1, and how, requires more detailed research.

Our study had some limitations: we did not identify (1) the precise sites facilitating the interaction of VIM and α-TAT1, (2) how viral IBs interact with α-TAT1 to increase the acetylation of α-tubulin, and (3) how VIM specifically degrades α-TAT1 through autophagy. Some researchers found that α-TAT1 was transported together with lysosomes in cells [43]. Therefore, α-TAT1 may have a relationship with lysosomes, and its degradation may be related to the regulation of autophagy by VIM [44–46]. There are few research articles on the roles of α-TAT1 in the formation of viral factories, but there are some reports concerning α-TAT1 and viral immune escape [42,47,48]. In future research, we will focus on the relationship between α-TAT1 and viral IBs, and clarify the degradation mechanism of α-TAT1 with VIM.

**Fig 7:**
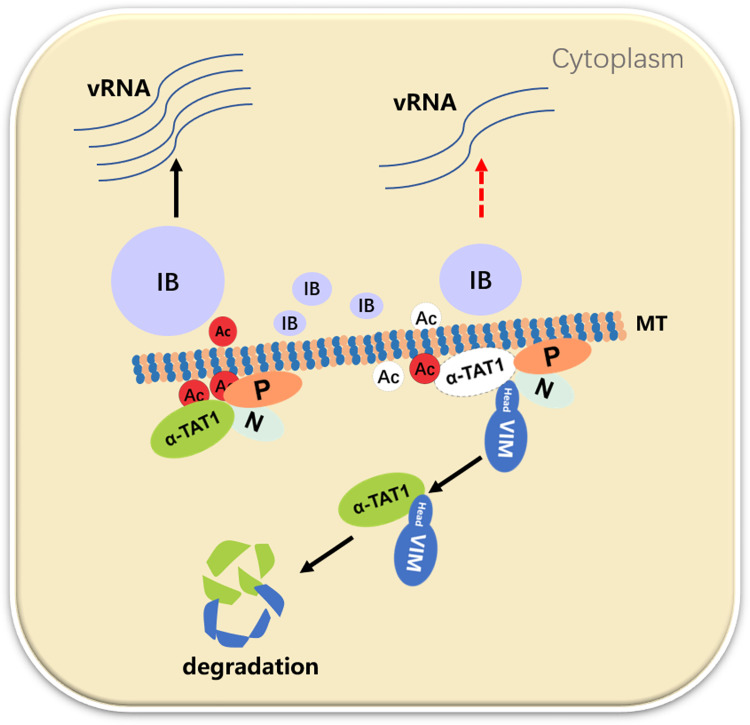
Model of VIM’s inhibitory effect on the fusion of HPIV3 IBs and viral replication.

To our knowledge, this is the first work to show that VIM can inhibit the acetylation of α-tubulin by enhancing α-TAT1 degradation in response to HPIV3 infection, and that VIM can also bind to components of the viral replication factory, inhibiting viral factory fusion, which, in turn, inhibits viral replication (as shown in Fig 7).

## Material and methods

### Cells, viruses, and reagents

HEK293T (Human embryonic kidney 293 cells were obtained from China Center for Type Culture Collection), HeLa (Human cervical cancer epithelial cells and were obtained from China Center for Type Culture Collection), A549 (Human non-small-cell lung carcinoma epithelial cells, obtained from China Center for Type Culture Collection), LLC-MK2 (monkey kidney cell line and were obtained from China Center for Type Culture Collection). Cells were cultured in Dulbecco’s modified Eagle’s medium (DMEM, Gibco) supplemented with 10% fetal bovine serum (FBS, Gibco) and 1% penicillin-streptomycin (Gibco). VIM stable knockdown cells (Hela-KD-VIM, A549-KD-VIM) were constructed via transduction with lentiviral shRNA transduction particles and cultured with puromycin (1 ug/ml, Sigma). VIM knock out cells (Hela-KO-VIM, A549-KO-VIM) were constructed via transfection with PX459 gRNA plasmid and monoclonal screening with puromycin (1 ug/ml, Sigma). Recombinant HPIV3 carrying an HA tag fused with the N-terminal of viral P (HPIV3_HA-P_) was constructed by our laboratory as described previously [45]. Multiplicity of infection (MOI = 0.1) of HPIV3 and HPIV3_HA-P_ infect LLC-MK2 cell for virus amplification. hRSV (MOI = 0.1) was amplificated by infecting Hep2 cell.

### Virus infection and titration

Cells cultured in 6-well, 24 well plates at a density of 70%-80% at 37°C/5% CO_2_ overnight were incubated with HPIV3 at an MOI of 1 plaque-forming unit/cell for 2h. Then fresh medium with 10% FBS replace the medium and cells cultures for another 24h or 36h. For plaque assay, additional 3–4 days are need for visible plaque forming. After being stained with crystal violet, the plaque was counted to calculate the viral titers. TCID50 assay was performed in 96 well plates with monolayer cells and the numbers of wells with cytopathic effect (CPE) in each dilution were counted. According to the Karber method, viral titers were calculated.

### Co-Immunoprecipitation and Western blot

Cells were harvested and lysed with lysis buffer (150 mM NaCl, 50mM Tris-HCl [pH 7.4], 1% Triton X-100, 1mM EDTA [pH 8.0] and 0.1% SDS, 0.1% protease inhibitor cocktail (MCE) for 30 min on ice. The supernatants were collected via centrifugation at 12000 g at 4°C for 30 min. Samples were boiled with SDS- PAGE loading buffer at 100°C for 10min. For immunoprecipitation, cells overexpressed N, P, VIM, VIM mutants and α-TAT1 were lysed and the lysates supernatants were collected and precleared by incubated with protein G Sepharose 4 Fast Flow beads for 1h at 4°C with rotation. After centrifugation, beads with specific primary antibodies were added in supernatants and incubated overnight at 4°C with rotation. Beads were collected and washed three times with washing buffer (5% [wt/vol] sucrose, 5 mM Tris-HCl [pH 7.4], 5 mM EDTA [pH 8.0], 500 mM NaCl, 1% [vol/vol] Triton X-100). Then the beads were boiled at 100°C for 10 min in 1× SDS protein loading buffer and analyzed by WB. Samples were resolved via 10% or 13% SDS polyacrylamide gel electrophoresis (SDS-PAGE). Nitrocellulose membranes with transferred proteins were blocked with 5% milk for 30min. Primary antibodies dilute in 1% milk were added and incubate with NC membrane overnight and then after three times wash with PBST (1×PBS, 0.1% Tween20) secondary antibodies were added for another 1h. Primary antibodies used were as follows: mouse anti-HN (Abcam), goat anti HPIV3 (Abcam), mouse anti-acetylation-α-tubulin (Santa Cruz), mouse anti α-tubulin (Abclonal), mouse anti-vimentin (Abcam), rabbit anti-vimentin (Abclonal), mouse anti-α-TAT1 (Abcam), mouse anti-PCNA (Santa Cruz), mouse anti-NPM (Santa Cruz), mouse anti-β-tubulin (Santa Cruz), rabbit anti-calnexin (sigma), rabbit anti-PDI (CST), rabbit anti-LC3B (CST). Mouse anti-c-Myc tag affinity gel (Biolegend) and mouse anti-Flag tag affinity gel (Biolegend), mouse anti-HA tag affinity gel (Dia-an biotech) were used in IP assays.

### Immunofluorescence assay

For immunofluorescence, HeLa cells were cultured on coverslips in 24 well plates overnight and then transfected with N, P, VIM and VIM mutant plasmids. HPIV3 and HPIV3_HA-P_ were added before transfected. Cells were harvested at the indicated times. 4% paraformaldehyde in 1×PBS was used to fix cells and 0.2% Triton X-100 were added for 20min at room temperature. After being blocked with 3% bovine serum albumin (BSA), primary antibodies diluted in 1% BSA were added and then secondary antibodies diluted in 1% BSA were added at 4°C.The primary antibodies used here were as follows: goat anti-HPIV3 (Abcam), rabbit anti-Flag tag (CST), rat APC-anti-Flag (Biolegend), mouse anti-c-Myc tag (MBL), mouse anti-HA tag (sigma), rabbit anti-HA tag (CST).The secondary antibodies used here were as follows: Alexa Fluor 488 donkey anti-rabbit IgG (Invitrogen), Alexa Fluor 594 donkey anti-mouse IgG (Invitrogen), Alexa Fluor 647 donkey anti-goat IgG (Invitrogen).Image J software were used to assay the fluorescence intensity of the cells in drawing boxes and intensity values were used. In order to visualizing the IBs in cells, HPIV3_HA-P_ virus was used and the location of HA-P was considered as viral IBs. HA-P and Myc-N co-expression also formed viral IBs in cells without HPIV3 infection. To counting the numbers of large, medium and small viral IBs, almost 30 cells in each group with IBs were counted.

### *In vitro* Luciferase assay

HPIV3 minigenome replicon system was described previously [6]. pGAD-P, pCDNA3.0-N-Myc, pGEM4-L, pOCUS-MG_HPIV3_, pCAGGS-T7 plasmids were laboratory saved. vTF7-3 virus with a recombinant T7 polymerase was used. Briefly, HeLa cell grown in 12 well plate was infected with vTF7-3 virus (MOI = 0.1) and pGAD-P, pCDNA3.0-N-Myc, pGEM-L, pOCUS-MG_HPIV3_ were transfected using lipo2000 reagent (Thermo Fisher). 24 hours later, cells were cracked by using lysis buffer (Promega). The firefly luciferase activity was assayed according to the firefly luciferase activity assay kit (Promega).

### Live cell imaging

HeLa cell stable expressed GFP-P_HPIV3_ was transfected with pCAGGS-mCherry-VIM after HPIV3 infection. About 30h latter, microtubule was stained by Microtubule -Tracker Deep Red Kit according to the manual (Beyotime). LSM 800(Zeiss) live cell imaging system was used to observe HPIV3 IBs and VIM with a temporal resolution of 3s per picture. In order to compare the effect of VIM on the fusion of inclusion bodies, we observed the process from approaching to fusion of inclusion bodies and calculated the time in live cell videos and showed representative pictures.

### HPIV3 binding and entry assay

According to a previous article [49], A549 WT/KO-VIM cells were infected with HPIV3 (MOI = 2) and incubated at 4°C for 30min with gentle rocking every 10min. Then the cells were washed three times with 1×PBS. For virus entry assay, the washed cells were overlaid with complete medium and incubated in 37°C/5% CO_2_ for 2h. Total RNA of WT/KO cells was extracted with Trizol reagent (Thermo Fisher) for RT-qPCR to compare the changes of viral RNA.

### RT-qPCR

Total RNA extracted was used to generate cDNA by using RT-qPCR maxima first strand cDNA synthesis master kit (Thermo Fisher). The qPCR analysis to measure the indicated RNA abundance. Relative abundance of the indicated RNA normalized to that of 18S RNA. The following primers were used:

HPIV3 L protein forward: 5’-AATAGCACAAACACGCGCAA-3’

HPIV3 L protein reverse: 5’-TGGGATTGTGTCCTGTGGTTT-3’

Human 18s RNA forward: 5’-GTAACCCGTTGAACCCCATT-3’

Human 18s RNA reverse: 5’-CCATCCAATCGGTAGTAGCG-3’

hRSV L protein forward: 5’- ACCGGGAATGTTCAGACAGG-3’

hRSV L protein reverse: 5’- GCAGTTCATCCAGCACATCAC-3’

### Peptides and peptides antiviral assay

20 full_TAT_ from VIM head domain 61–80 amino acids were synthesized (Sangong) as follow:

TAT-GSG-YATRSSAVRLRSSVPGVRLL

TAT sequence: YGRKKRRQRRR, ‘GSG’ is the linker.

TAT and 20 full_TAT_ were diluted in PBS filtrated by 0.22μm filter. After HPIV3 infection, peptides were added to the culture medium containing 5% FBS for 24 hours, and then cells were lysis for viral protein level detection or treated with Trizol to obtain the total RNA for viral RNA level detection.20 full was mixed with TAT peptide and transferred to the cell culture medium with HPIV3_HA-P_ infected cells to validate the colocalization with viral IBs, and a no viral infection control was applied. Then cells on the cover slip were used for Immunofluorescence assay.

### gRNA sequences and shRNA sequence

For VIM knockout cell lines:

gRNA1#: 5’- CAACGACAAAGCCCGCGTCG-3’

gRNA2#: 5’- TCCTACCGCAGGATGTTCGG -3’

gRNA3#: 5’-CACCGATGCGCCTCCGGGAGAAGTA-3’

shRNA1#: 5’-GCAGGATGAGATTCAGAATAT-3’

shRNA2#: 5’-GACAGGTTATCAACGAAACTT-3’

gRNAs plasmids were generated by using pX459 backbone. shRNAs plasmids were generated by using PLKO.1 backbone. pSPAX and pMD2.G were used to package lentiviruses.

For α-TAT1 knockout A549 cell line (also VIM-KO):

gRNA: 5’-GATCGTGAGGCTCATAATG-3’

PX458 backbone with GFP tag was used to generate gRNA plasmid. A549 cells (KO-VIM) were transfected with this plasmid by using lipo2000. After 24 hours, cells were digested into individual cells and sorted by flow cytometry to collect GFP positive cells, and they were considered as a pool of α-TAT1 knockout cells.

## Supporting information

S1 Fig Preformed HPIV3 N–P complex could not interact with overexpressed VIM protein. (A) The N–P complex did not interact with VIM when they were mixed in vitro. HA–P, N-Myc, and Flag–VIM plasmids were transfected separately into HEK293T cells. HA-P and N-Myc were co-transfected to form the N–P complex. The lysates were mixed as follows: N with P, N with VIM, P with VIM, N with P and VIM, and N–P complex with VIM. The lysates were immunoprecipitated using anti-c-Myc tag affinity gel. Proteins were detected using corresponding antibodies. (B) VIM did not interact with the preformed N–P complex when they were mixed. N-Myc, HA–P, and Flag–VIM plasmids were transfected separately in HEK293T cells. N-Myc and HA–P were co-transfected into HEK293T cells or with Flag–VIM. Cell lysates were mixed as follows: N with P, VIM with P, VIM with N, and VIM with N–P complex. Cell lysates were immunoprecipitated using anti-FLAG tag affinity gel. The results clearly showed that the preformed N–P complex could not interact with VIM.
(TIF)

S2 Fig Construction and detection of stable-knockdown and VIM-knockout cell lines. (A) Transient transfection of sgRNA plasmids with gRNA-targeted VIM gene in HeLa cells for verification of the effects. An empty pX459 vector was used as a control. (B) The protein levels of VIM in VIM-stable-knockdown HeLa/A549 cell lines compared with VIM in WT cells are shown by using specific antibodies. (C) Five monoclonal VIM-KO cells of HeLa and A549 cell lines. Their VIM expression levels are shown, along with those in wild-type cells. We selected KD-VIM1# and KO-VIM1# for our experiments involving these cell lines.
(TIF)

S3 Fig VIM enhances the degradation of α-TAT1 and suppresses the acetylation of α-tubulin to inhibit the fusion of HPIV3 IBs. (A) HPIV3 infection caused VIM to aggregate. HeLa cells were infected with HPIV3 for 24 h (MOI = 0.1), goat anti-HPIV3 and mouse anti-VIM were used; scale bar: 10 μm. (B) HPIV3 IBs co-located with VIM and microtubules, and VIM suppressed the fusion of HPIV3 IBs. HeLa-GFP–P cells infected with HPIV3 (MOI = 0.1) and transfected with mCherry–VIM plasmid and microtubules were labeled with paclitaxel. Scale bar: 2 μm (S2 Movie and S3 Movie). The white arrow shows the fusion of IBs, and the black arrow shows the IBs with VIM and microtubules. (C) IB fusion time statistics corresponding to (B). Individual data represent the IB contact and fusion times. Numbers of IBs adjacent to microtubules or to both mCherry–VIM and microtubule. The GFP–P indicated the presence of viral IBs. IBs were calculated according to their size (large, medium, or small). The analysis results in (C) correspond to (B) and other fields. Panel B shows one of the fields we used for analysis. (D) VIM enhanced α-TAT1 degradation through an autophagy-related pathway. Rapamycin (100 μM, Sigma), CQ (50 nM, Sigma), BafA1 (100 nM, MCE), and MG132 (20 nM, MCE) were used to detect the major degradation pathways of α-TAT1. Rapamycin and MG132 enhanced α-TAT1 degradation (lanes 3 and 6). BafA1 inhibited α-TAT1 degradation (lane 5). Proteins were expressed in HeLa cells and detected by using specific antibodies. Cellular LC3I/II levels were considered markers of the level of autophagy. (E) The formation of HPIV3_HA-P_ IBs in KO-α-TAT1 cell pools generated from KO-VIM A549 cells, and WT, KO-VIM A549 cells were also tested. Acetylated α-tubulin were detected by using specific antibody. Values are means ± SDs from three experiments. Student’s t test: * p value<0.05, ** p value<0.01, and ns = not significant.
(TIF)

S4 Fig Tests of the effects of TAT and 20 full_TAT_ peptides on cell viability and antiviral ability. (A) FAM-20 full peptide in HeLa cells with or without HPIV3 infection. HeLa cells were infected with HPIV3_HA-P_, and then, peptides were mixed and cocultured with the cells. Rabbit anti-HA antibody was used. Scale bar:10 μm. (B, C) Cell viability was not affected by the peptide treatment. HeLa cell viability was tested using CCK8 after 20full_TAT_ and TAT peptides were applied for treatment according to the manufacturer’s manual. Data were normalized and analyzed. (D, E) The HPIV3 viral RNA level was affected by 20 full_TAT_ peptide. HPIV3 RNA level was assayed using RT-qPCR after the TAT and 20 full_TAT_ peptides were applied for treatment. In panels B, C, D, and E, the values are means ± SDs from three experiments. Student’s t test: * p value<0.05. ns = not significant.
(TIF)

S1 Movie The distribution of HPIV3 IBs in HeLa-GFP–P cells. This movie is as shown in Fig 3E. The mCherry–VIM plasmid was transfected into HeLa-GFP–P cells, and the cells were infected with HPIV3. After 24 hours, cells infected with HPIV3 and expressing mCherry–VIM were used for live cell imaging. The image showed the fused cells infected by HPIV3. Area 1 includes mCherry–VIM and GFP–P-labeled IBs. Area 2 is an area without mCherry–VIM. Scale bar: 5 μm.
(AVI)

S2 Movie HPIV3 IBs adjacent to microtubules in HeLa-GFP–P cells without mCherry–VIM expression were visualized by live cell imaging. This movie is as shown in the first row of S3B Fig. Microtubules were labeled using paclitaxel. GFP–P-labeled HPIV3 IBs were shown with microtubules. Scale bar: 2 μm.
(AVI)

S3 Movie HPIV3 IBs adjacent to microtubules and mCherry–VIM were visualized by live cell imaging. This movie is as shown in the second row of S3B Fig. The microtubule was labeled using paclitaxel. Cells were transfected with mCherry–VIM. GFP–P-labeled HPIV3 IBs were shown with microtubules and mCherry–VIM. Scale bar: 2 μm.
(AVI)
