# Supplementary figures and images for: Vimentin inhibits α-tubulin acetylation via enhancing α-TAT1 degradation to suppress the replication of human parainfluenza virus type 3
Source: PLoS Pathog. 2022 Sep 15;18(9):e1010856. doi: 10.1371/journal.ppat.1010856 (PMC9524669; doi:10.1371/journal.ppat.1010856)

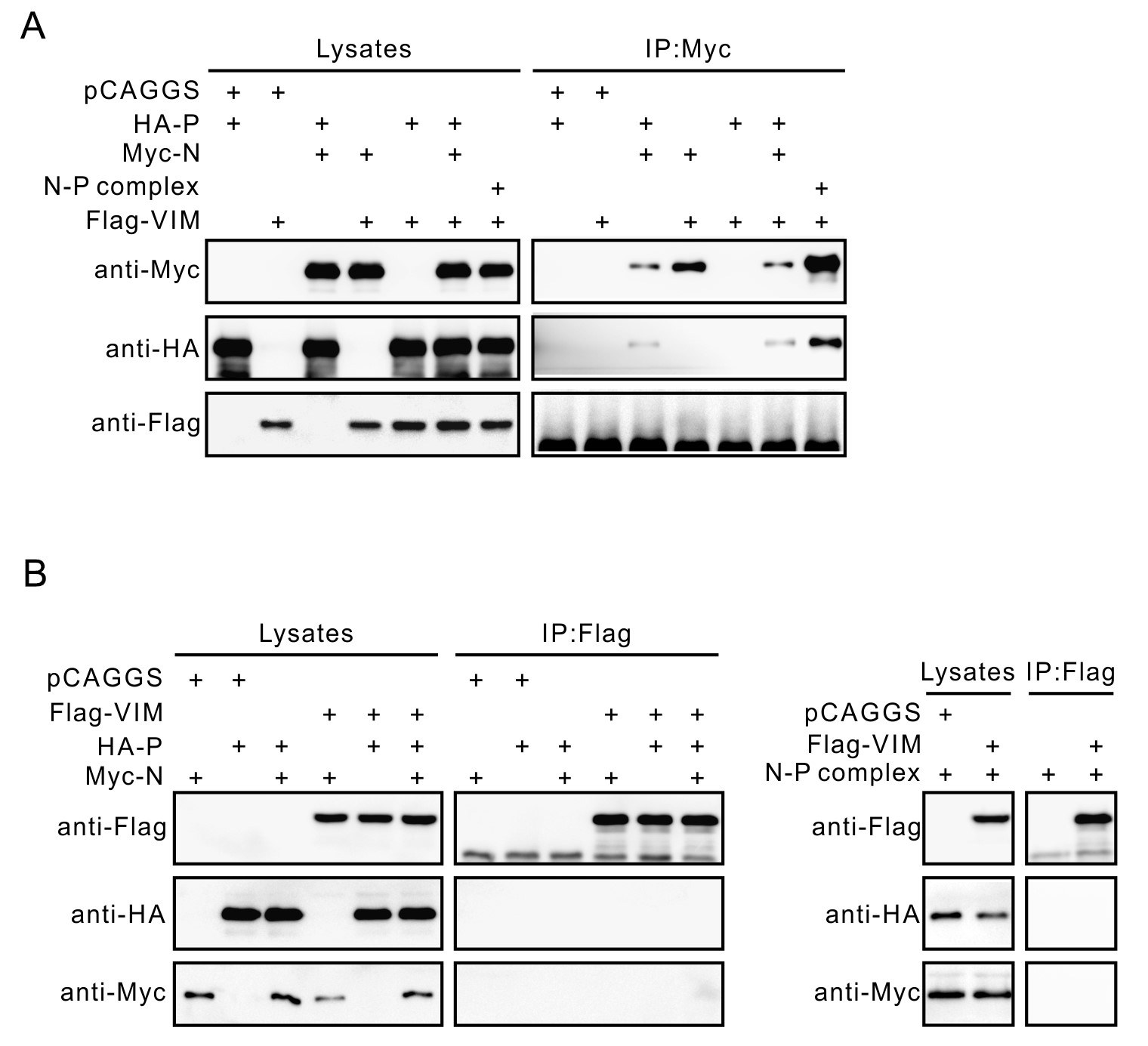

Supplement: S1 Fig — (A) The N–P complex did not interact with VIM when they were mixed in vitro. HA–P, N-Myc, and Flag–VIM plasmids were transfected separately into HEK293T cells. HA-P and N-Myc were co-transfected to form the N–P complex. The lysates were mixed as follows: N with P, N with VIM, P with VIM, N with P and VIM, and N–P complex with VIM. The lysates were immunoprecipitated using anti-c-Myc tag affinity gel. Proteins were detected using corresponding antibodies. (B) VIM did not interact with the preformed N–P complex when they were mixed. N-Myc, HA–P, and Flag–VIM plasmids were transfected separately in HEK293T cells. N-Myc and HA–P were co-transfected into HEK293T cells or with Flag–VIM. Cell lysates were mixed as follows: N with P, VIM with P, VIM with N, and VIM with N–P complex. Cell lysates were immunoprecipitated using anti-FLAG tag affinity gel. The results clearly showed that the preformed N–P complex could not interact with VIM. (TIF) [file ppat.1010856.s001.tif]

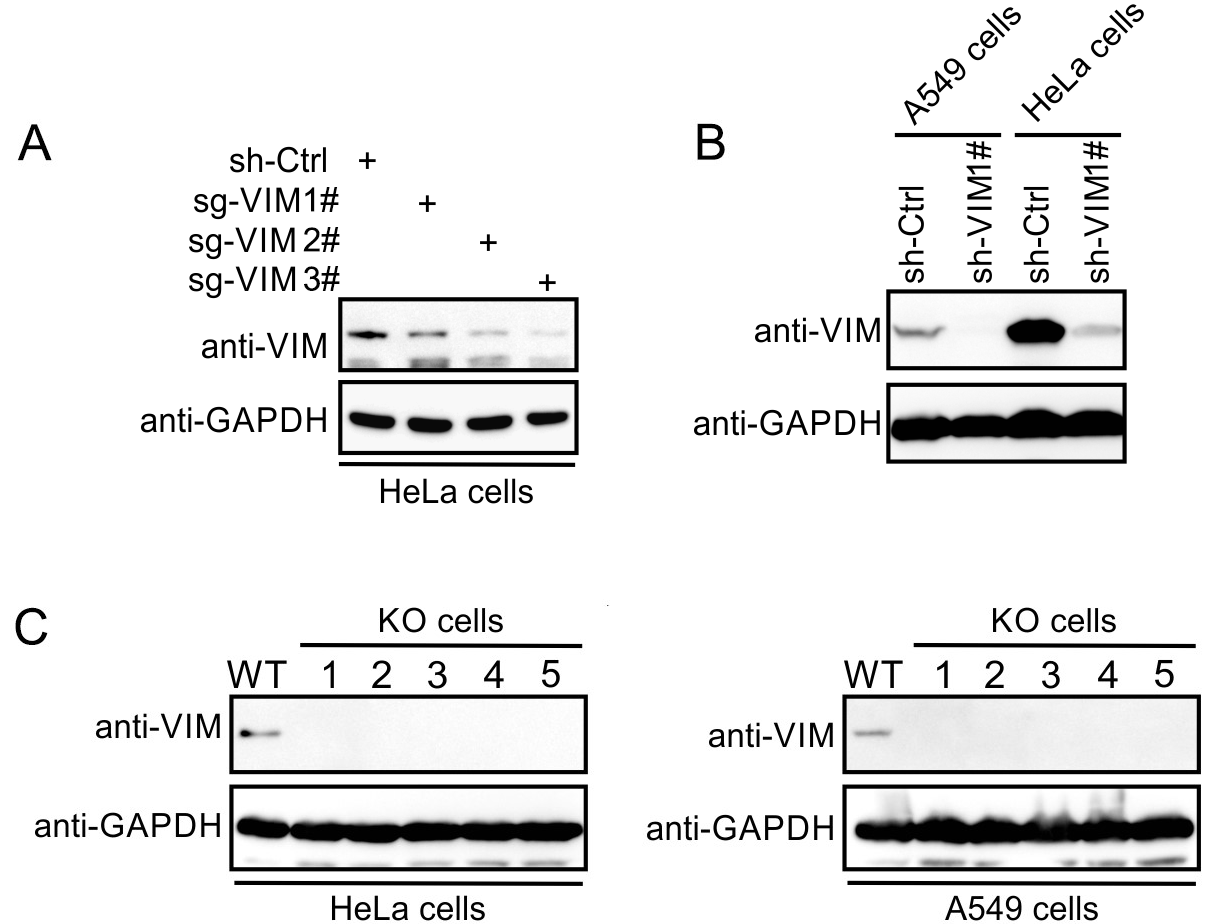

Supplement: S2 Fig — (A) Transient transfection of sgRNA plasmids with gRNA-targeted VIM gene in HeLa cells for verification of the effects. An empty pX459 vector was used as a control. (B) The protein levels of VIM in VIM-stable-knockdown HeLa/A549 cell lines compared with VIM in WT cells are shown by using specific antibodies. (C) Five monoclonal VIM-KO cells of HeLa and A549 cell lines. Their VIM expression levels are shown, along with those in wild-type cells. We selected KD-VIM1# and KO-VIM1# for our experiments involving these cell lines. (TIF) [file ppat.1010856.s002.tif]

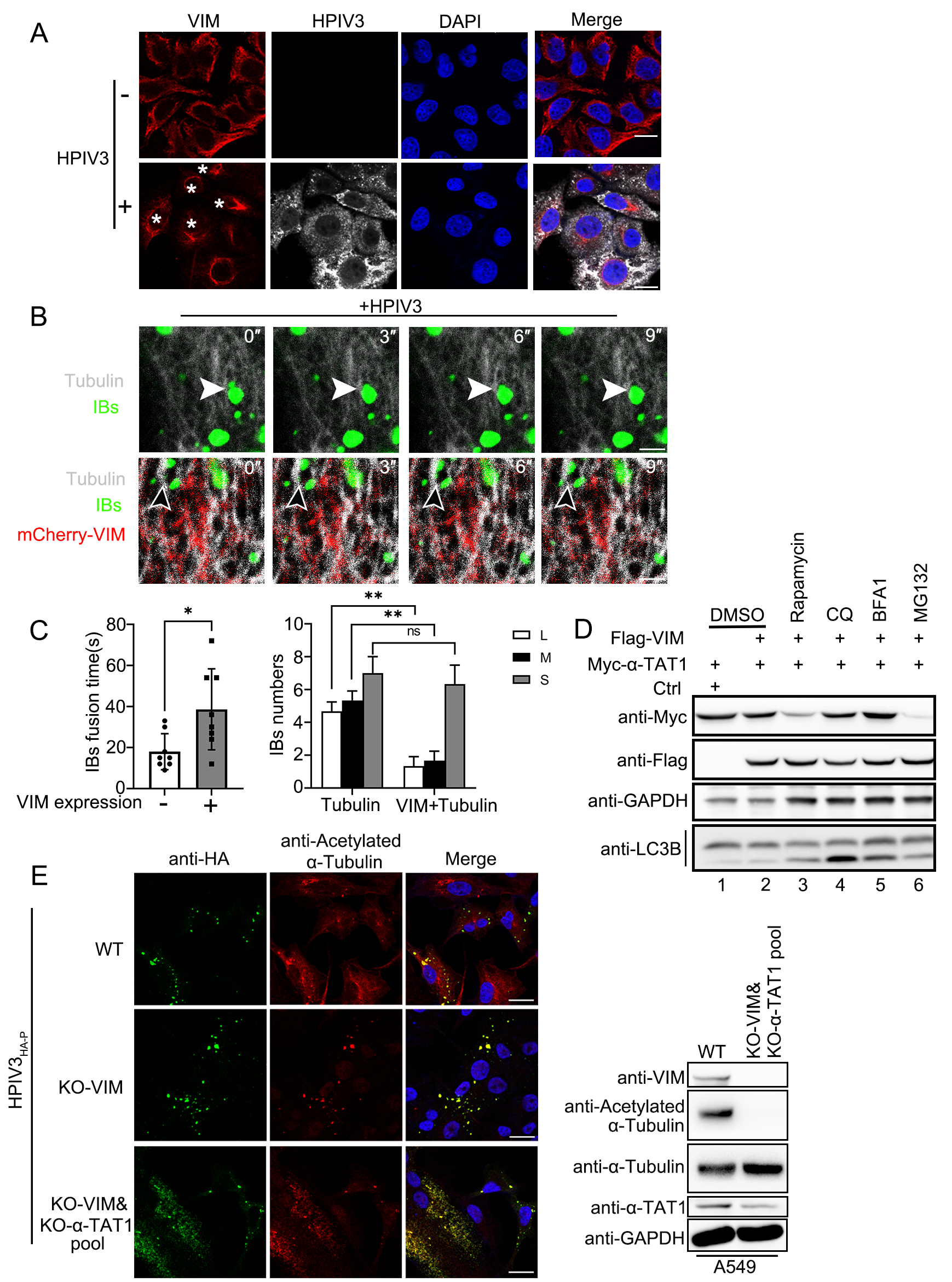

Supplement: S3 Fig — (A) HPIV3 infection caused VIM to aggregate. HeLa cells were infected with HPIV3 for 24 h (MOI = 0.1), goat anti-HPIV3 and mouse anti-VIM were used; scale bar: 10 μm. (B) HPIV3 IBs co-located with VIM and microtubules, and VIM suppressed the fusion of HPIV3 IBs. HeLa-GFP–P cells infected with HPIV3 (MOI = 0.1) and transfected with mCherry–VIM plasmid and microtubules were labeled with paclitaxel. Scale bar: 2 μm (S2 Movie and S3 Movie). The white arrow shows the fusion of IBs, and the black arrow shows the IBs with VIM and microtubules. (C) IB fusion time statistics corresponding to (B). Individual data represent the IB contact and fusion times. Numbers of IBs adjacent to microtubules or to both mCherry–VIM and microtubule. The GFP–P indicated the presence of viral IBs. IBs were calculated according to their size (large, medium, or small). The analysis results in (C) correspond to (B) and other fields. Panel B shows one of the fields we used for analysis. (D) VIM enhanced α-TAT1 degradation through an autophagy-related pathway. Rapamycin (100 μM, Sigma), CQ (50 nM, Sigma), BafA1 (100 nM, MCE), and MG132 (20 nM, MCE) were used to detect the major degradation pathways of α-TAT1. Rapamycin and MG132 enhanced α-TAT1 degradation (lanes 3 and 6). BafA1 inhibited α-TAT1 degradation (lane 5). Proteins were expressed in HeLa cells and detected by using specific antibodies. Cellular LC3I/II levels were considered markers of the level of autophagy. (E) The formation of HPIV3HA-P IBs in KO-α-TAT1 cell pools generated from KO-VIM A549 cells, and WT, KO-VIM A549 cells were also tested. Acetylated α-tubulin were detected by using specific antibody. Values are means ± SDs from three experiments. Student’s t test: * p value<0.05, ** p value<0.01, and ns = not significant. (TIF) [file ppat.1010856.s003.tif]

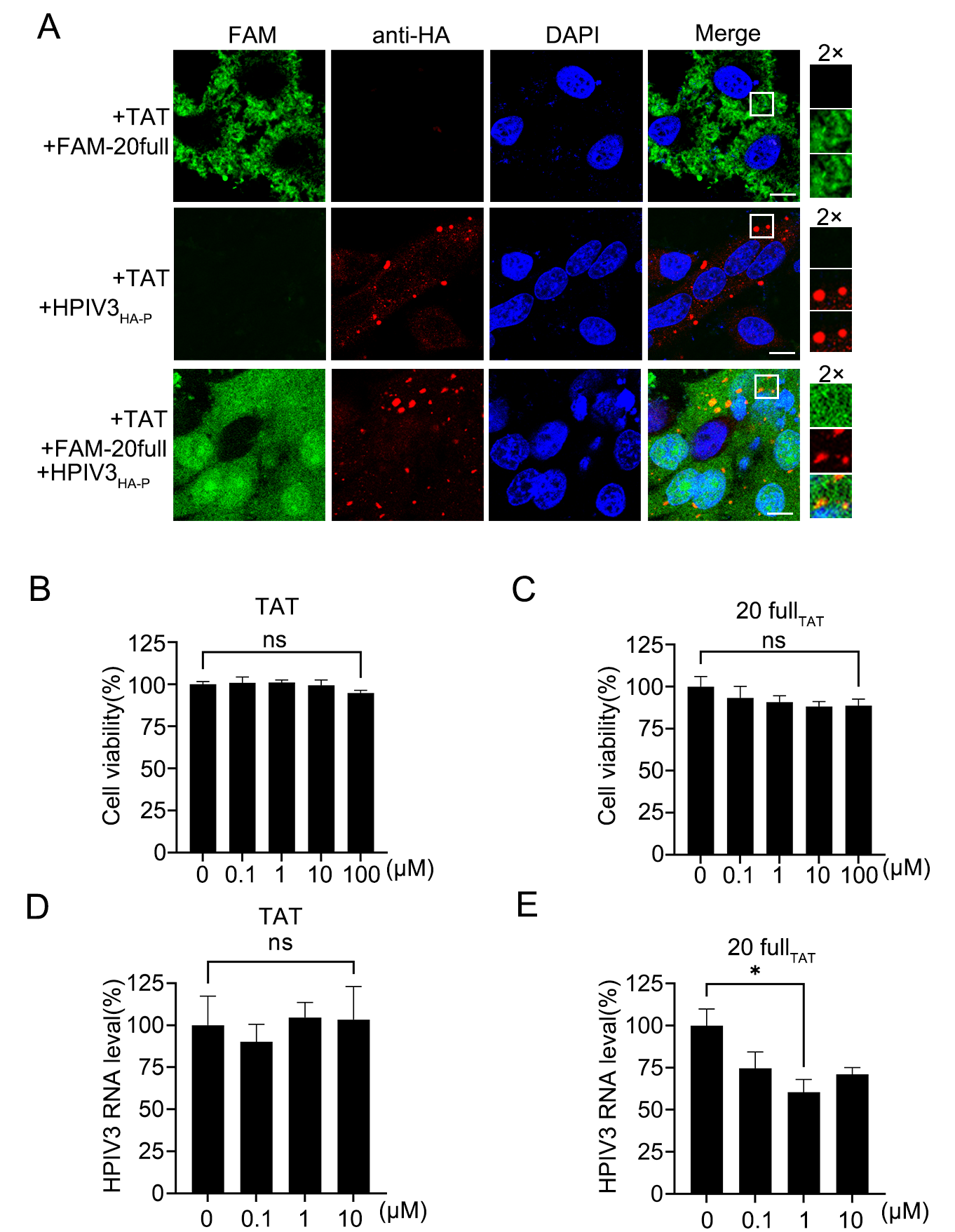

Supplement: S4 Fig — (A) FAM-20 full peptide in HeLa cells with or without HPIV3 infection. HeLa cells were infected with HPIV3HA-P, and then, peptides were mixed and cocultured with the cells. Rabbit anti-HA antibody was used. Scale bar:10 μm. (B, C) Cell viability was not affected by the peptide treatment. HeLa cell viability was tested using CCK8 after 20fullTAT and TAT peptides were applied for treatment according to the manufacturer’s manual. Data were normalized and analyzed. (D, E) The HPIV3 viral RNA level was affected by 20 fullTAT peptide. HPIV3 RNA level was assayed using RT-qPCR after the TAT and 20 fullTAT peptides were applied for treatment. In panels B, C, D, and E, the values are means ± SDs from three experiments. Student’s t test: * p value<0.05. ns = not significant. (TIF) [file ppat.1010856.s004.tif]
